# Translating electronic health record-based patient safety algorithms from research to clinical practice at multiple sites

**DOI:** 10.1136/bmjhci-2022-100565

**Published:** 2022-07-14

**Authors:** Andrew J Zimolzak, Hardeep Singh, Daniel R Murphy, Li Wei, Sahar A Memon, Divvy K Upadhyay, Saritha Korukonda, Lisa Zubkoff, Dean F Sittig

**Affiliations:** 1 Center for Innovations in Quality, Effectiveness and Safety, Michael E DeBakey VA Medical Center, Houston, Texas, USA; 2 Department of Medicine, Baylor College of Medicine, Houston, Texas, USA; 3 Division of Quality, Safety and Patient Experience, Geisinger, Danville, PA, USA; 4 Research Institute, Geisinger, Danville, PA, USA; 5 Geriatric Research Education and Clinical Center, Birmingham VA Medical Center, Birmingham, Alabama, USA; 6 Division of Preventive Medicine, The University of Alabama at Birmingham, Birmingham, Alabama, USA; 7 School of Biomedical Informatics, The University of Texas Health Science Center at Houston, Houston, Texas, USA

**Keywords:** Medical Informatics, Electronic Health Records, Health Services Research, Patient Care

## Abstract

**Introduction:**

Researchers are increasingly developing algorithms that impact patient care, but algorithms must also be implemented in practice to improve quality and safety.

**Objective:**

We worked with clinical operations personnel at two US health systems to implement algorithms to proactively identify patients without timely follow-up of abnormal test results that warrant diagnostic evaluation for colorectal or lung cancer. We summarise the steps involved and lessons learned.

**Methods:**

Twelve sites were involved across two health systems. Implementation involved extensive software documentation, frequent communication with sites and local validation of results. Additionally, we used automated edits of existing code to adapt it to sites’ local contexts.

**Results:**

All sites successfully implemented the algorithms. Automated edits saved sites significant work in direct code modification. Documentation and communication of changes further aided sites in implementation.

**Conclusion:**

Patient safety algorithms developed in research projects were implemented at multiple sites to monitor for missed diagnostic opportunities. Automated algorithm translation procedures can produce more consistent results across sites.

## Introduction

Health information technology shows promise for improving patient safety. Electronic health record (EHR) data are increasingly available and can prevent or detect potential patient safety events,^1^ thus providing knowledge to promote safety, learning and improvement. We previously developed electronic trigger (e-trigger) tools that query EHR databases to identify potential delays in follow-up of abnormal tests.^2^ Such algorithms can identify when a laboratory or radiology report suggests the need for additional testing, but appropriate follow-up has not occurred.^3^

**Figure 1:**
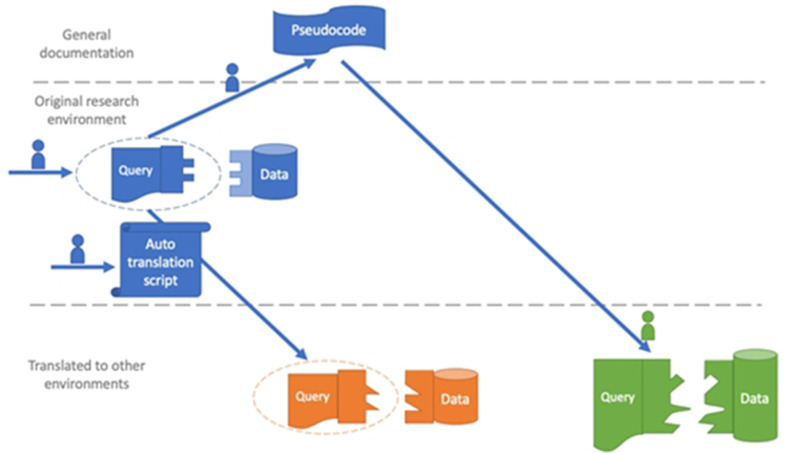
Workflow of code translation from the research environment to multiple operational environments. In prior work, one team member developed structured query language algorithms to retrieve potential missed cancer follow-up cases from the Veterans Affairs (VA) research data warehouse (blue). For the present study, the team developed a script to translate the algorithms automatically to the VA operational data warehouse (orange), which is structured differently. The team also created pseudocode and documentation so that the algorithms could be translated to non-VA data warehouses (green), which have very different structures from VA.

Patient safety algorithms developed through research must be implemented in clinical practice.^4^ However, there are no well-defined methods for implementation, and most studies do not make computer code available after publication,^5 6^ limiting opportunities to use algorithms clinically. Sharing code would improve replication, implementation and return on investment for research funding. A typical approach to reusing computer code in different institutions is to adapt each institution’s data to a common data model (CDM).^7^ Still, researchers invest much effort into algorithms that do not use CDMs. We believe that another alternative may advance the field: translate code and send it to sites with a supplemental description (figure 1).

We describe how researchers collaborated with multiple clinical sites to implement two algorithms that identify patients without timely follow-up of abnormal test results, warranting evaluation for lung or colorectal cancer. We also describe lessons learnt from the process.

## Methods

### Baseline algorithms

We aimed to translate existing structured query language (SQL) code developed during Veterans Affairs (VA) research to improve healthcare delivery inside and outside VA. Our prior work developed two algorithms that identify potential delayed follow-up of tests suggesting lung or colorectal cancer.^3 8^ In brief, the code contains value sets for three steps: (a) retrieve records with tests (blood, stool, imaging) concerning for cancer, (b) exclude records where follow-up is unnecessary or with known causes for abnormalities, (c) exclude records with appropriate follow-up. Extending this work, the current project demonstrates successful implementation at 11 VA sites and Geisinger, a large health system in Pennsylvania. Although VA has a national database, we sent code to VA sites rather than analyse their data because each hospital knows best how to assess its safety challenges, and regulations separate research from operational data. Baylor College of Medicine and Geisinger institutional review boards approved the work (protocol H-45450).

### Barriers

Translation required overcoming several barriers: (1) tables are named differently in VA operational and research databases, (2) need for ease of use for sites with varying experience, (3) VA operational database has stricter user permissions, requiring extensive changes to techniques for storing intermediate and final results and (4) need to adapt to non-VA sites.

### Code translation/implementation

We wrote two Python scripts that edit SQL, automatically renaming tables using operational conventions (barriers 1 and 3). We also enhanced code usability and documentation (barriers 2 and 4). For instance, the original programmer reorganised code (eg, collecting user-defined settings together) and we drafted documentation and pseudocode (human-readable description outlining code steps to guide non-VA implementers: see online supplemental file 1). These improvements were informed by questions from sites reviewing code and documentation. Figure 1 shows a process overview. To track code and sites’ requests, we stored materials on a public GitHub repository with issue tracker (https://github.com/zimolzak/instruct-project-etrigger-sql). Finally, we scheduled didactic teleconferences and hosted office hours every 1–2 weeks to answer questions.

10.1136/bmjhci-2022-100565.supp1 Supplementary data

Implementation at VA proceeded as a stepped wedge, with three cohorts, 3–4 sites per cohort, and a 3-month ‘prework’ phase to improve code familiarity. Geisinger implemented as a single site (e-trigger applied to all locations in the system). Site clinicians validated a sample of retrieved charts. All sites reviewed positive cases, but not all reviewed negative cases.

## Results

### Technical

The automated script made extensive changes (30% of e-trigger code). During validation, there were 107 further code changes from 2019 to 2021. Most changes generalised to all sites (eg, expanding documentation, improving usability, improving interpretability). Site-specific changes included VA sites wishing to focus only on only one clinic among several in their city/region.

### Workflow

Our centralised code adaptation saved each site from performing multiple edits (over ten large find-and-replace operations per algorithm), thus reducing work and potential errors. Estimated time saved ranges from 1 to 6 hours per site.

### Outcomes

All sites successfully ran the e-triggers. Validation revealed that all cases were retrieved appropriately. False positives fell into previously described categories,^3 8^ for example, patients declining follow-up. We observed a trend towards more outside cancer care at Geisinger (eg, initial cancer diagnosis made elsewhere, before first Geisinger visit).

### Support

From November 2019 to February 2022, we logged 66 e-mail conversations among all sites (average 5.5 per site), plus estimated 1 hour live discussion per site. Topics included modifying e-trigger time frames, database errors and anomalous results (eg, zero tests found). Troubleshooting occurred predominantly over e-mail, and teleconferences focused on intensive troubleshooting. We anecdotally observed that required preparation time decreased as implementation progressed through VA cohorts, although we did not measure this directly.

## Discussion

We successfully translated two patient safety algorithms from research to practice in multiple clinical sites, using a new approach: large-scale automated code translation rather than the typical method using a CDM.^7^ Lessons learnt include:

1. Write pseudocode with a complete value set listing for organisations with different data models.
2. Use source code control such as Git. Make code open to all sites.
3. Communicate frequently with sites receiving code.
4. Clinical personnel at each site should validate results.

Our approach is valuable when a research algorithm uses a non-standard data model; others can use the algorithm after translation to a new model. We expect centralised edits to decrease risk of errors and inconsistencies. Sending code to individual sites allows healthcare operations to benefit from our algorithms for missed tests concerning for cancer, by finding individual high-risk patients, notifying providers or measuring quality in a population. Apart from business reasons, there are scientific reasons for code sharing.^9^ A paper’s reviewers and readers should have access to the authors’ code to replicate the study, which they likely could not do from the methods section alone. Despite the push for research code sharing, a 2019 review showed 0 of 194 studies made analysis scripts available.^5^ Another showed that most studies decline to submit statistical code to a journal, or they include minimal documentation.^6^ Our online supplemental file 1 description is similar to the approach of Phenotype KnowledgeBase, a resource for sharing electronic phenotypes,^10^ but our code translation approach is unique, and our use of pseudocode for systems with different data structures is a strength.

Our work has several limitations. The adaptations required by our sites may not be desired by others. Second, the script that edits SQL code would have to be rewritten for other codebases. Nevertheless, the methodology of a programme automatically modifying another programme would be transferable and still save time. Third, since our focus was implementation, we did not quantify the benefit of pseudocode by assessing sites’ implementation before and after pseudocode, but this could be a topic for future research.

## Conclusions

We describe a strategy to efficiently translate patient safety algorithms from research to practice in multiple health systems. We also provide generalisable lessons learnt. This approach impacts the care of individual patients, increases the return on investment of research funding, and potentially impacts long-term population health.

## Data Availability

All data relevant to the study are included in the article or uploaded as supplementary information.
