# Supplementary material for: Translating electronic health record-based patient safety algorithms from research to clinical practice at multiple sites
Source: BMJ Health Care Inform. 2022 Jul 14;29(1):e100565. doi: 10.1136/bmjhci-2022-100565 (PMC9289019; doi:10.1136/bmjhci-2022-100565)
Supplement: Supplementary data [file bmjhci-2022-100565supp001.pdf]

Cancer Test Result e-Trigger Manual

Guidance complied by: Sahar Memon, Andrew Zimolzak, Li Wei, Daniel Murphy, Hardeep Singh.

Affiliation: Michael E. DeBakey Veterans Affairs Medical Center, and Baylor College of Medicine. Houston, TX, USA.

For questions: zimolzak@bcm.edu and Sahar.Memon@bcm.edu

Date: October, 2021

Overview

This manual outlines procedures for implementing e-triggers that identify missed opportunities in follow-up of 'red flag' findings suspicious for colorectal or lung cancer. In general, each SQL file proceeds by:

1. defining the red flags that warrant additional evaluation for cancer (often labs or imaging),
2. excluding other explanations for the red flags, such as already diagnosed colon cancer, or known cause of bleeding in the upper GI tract (often based on ICD/CPT codes),
3. excluding patients for whom follow-up is not deemed necessary, and
4. excluding patients for whom appropriate follow-up was already done (e.g., lung biopsy, follow-up imaging, tumor board, usually based on stop codes and procedure codes).

As an example, the e-trigger for colorectal cancer identifies patients with positive fecal blood tests or iron deficiency anemia, and then excludes patients with any of the following: advanced age, deceased status, known colon cancer, prior colectomy, terminal illnesses or hospice care, presence of a known diagnosis that would cause bleeding in the upper GI tract rather than lower GI tract, and appropriate colonoscopy or GI referral.

This code is public domain for *anyone* to use as they wish. However, if you have a published paper based in part on the code, we ask that you cite:

Zimolzak AJ, *et al. BMJ Health Care Inform* 2022; 29:e100565. doi: 10.1136/bmjhci-2022-100565

- Development and Validation of Trigger Algorithms to Identify Delays in Diagnostic Evaluation of Gastroenterological Cancer. *Clin Gastroenterol Hepatol.* 2018 Jan;16(1):90-98.
- Computerized Triggers of Big Data to Detect Delays in Follow-up of Chest Imaging Results. *Chest.* 2016 Sep;150(3):613-20.
- Development and Validation of Electronic Health Record-based Triggers to Detect Delays in Follow-up of Abnormal Lung Imaging Findings. *Radiology.* 2015 Oct;277(1):81-7.
- Electronic health record-based triggers to detect potential delays in cancer diagnosis. *BMJ Qual Saf.* 2014 Jan;23(1):8-16.

How are the e-triggers designed?

The lung and colorectal e-triggers are examples of one type of e-trigger, which we call a *close the loop e-trigger.* These two e-triggers answer the question, "How do you find (in the database) patients who had a test that shows a possibility of cancer, but who have **not** had timely follow-up?"

Further details about exactly what constitutes exclusion or follow-up can be found in the e-trigger manual appendices. Non-VA sites should consult these appendices, as well as SQL code in order to reimplement the e-triggers in local SQL.

How to Apply e-Trigger Process at a VA Facility

Downloading the SQL code

1. The most recent version of the code can be downloaded from github.com/zimolzak/instruct-project-etrigger-sql where you can also find additional procedures for setting dates, and guidance about which tables to export for final reporting.
2. Before downloading, jot down or copy/paste the text in the bar near the top of GitHub, especially the **seven random-looking letters and numbers** such as "LWeiBCM Update Lung.sql … **8c2f54a** 2 days ago." This will identify the exact version of the code you downloaded, for future reference.
3. Click on the SQL file you want above (such as `Lung.sql`).
4. Click on the grey button "Raw" near the top the page that comes up.
5. Use your browser menu to save file to disk (such as "File / Save Page As…").

Setup

This example assumes that you want to retrieve one month worth of e-trigger counts and patient information.

1. Find the first day of the current month (e.g., if today is Feb 19, you rewind to find Feb 1).
2. Subtract two more months from that (so you get Dec 1) if you are running `Lung.sql`. Subtract *three months* if you are running `Fobt.sql`.
3. Set `sp_start` equal to that (such as `set @sp_start='2019-12-01 00:00:00'`).
4. Set `sp_end` to the end of that month (such as `set @sp_end='2019-12-31 23:59:59'`).
5. You need to set your sta3n and sta6a. You can do this by commenting/uncommenting code in lines 100–124 for `Lung.sql`, or lines 118–146 for `Fobt.sql`.
6. Done! Other variables like `fu_period` can be left as-is.

Running code

1. Start your operational access to the data warehouse via your usual method (e.g., desktop or Citrix connection to SQL Server Management Studio software). Login to a SQL server (e.g. `vhacdwa01.vha.med.va.gov`) and authenticate (using either username such as `vha01\vhabhs...` plus password, or using Windows authentication).
2. *Recommended:* Run sections of the SQL file sequentially (for example, lines 1–198 of `Fobt.sql` cover the first two `INSERT INTO` operations concerning tables that were newly created), inspecting for errors.

   Alternatively: run the query all at once, inspecting for errors.

Viewing and validating data

- To view patients with positive lung e-triggers, run the following SQL, after `Lung.sql` completes: `select * from #Lung_Sta3n528_3_Ins_U_TriggerPos`
- For lung counts, `Lung_Sta3n528_4_01_Count` should display automatically.
- To view patients with positive colorectal e-triggers, run the following SQL, after `Fobt.sql` completes: `select * from #FOBT_Sta3n528_5_Ins_U_TriggerPos`

- For colorectal counts, `FOBT_Sta3n528_5_Ins_X_count` should display automatically.

The site personnel doing **validation** should receive the "Ins_U_TriggerPos" tables (which will contain PHI, so don't send outside your station). You may review all, or randomly select a few patients with positive e-trigger, and securely transmit last name and last 4 of SSN from these patients to the reviewer, who will validate via CPRS that the sample patients have a positive red flag inside the time period of interest.

Final note: The VA Corporate Data Warehouse releases patch updates periodically, and this might require ongoing minor changes/updates to SQL code, by each site analyst. Standard codes (CPT, ICD, ICDProc, LOINC, Stop code, etc.) tend to change every year, with addition of new codes and removal of old codes. These changes require corresponding updates in the SQL code. Important note here is that you only add new codes to the SQL; do *not* remove the old ones (this is so the e-trigger continues to capture usage of both the historical and new codes).

Further reading (relevant files/attachments)

- Reducing Missed Test Results Change Package
- `Lung.sql` code file (see GitHub)
- `Fobt.sql` code file (see GitHub)

APPENDIX: Colorectal Red Flag Criteria

1. Identify all patient records with iron deficiency anemia, defined as:

(hemoglobin (Hb) less than or equal to 11 g/dL [1] *and* mean corpuscular volume (MCV) less than or equal to 81 fL [2] *and* no ferritin greater than or equal to 100 ng/mL within 12 months before or 60 days after CBC (i.e., ferritin not checked or result < 100) [3])

OR

(a positive fecal occult blood test (FOBT) or fecal immunochemical test (FIT) [4] result)

Clinical Exclusion Criteria

2. Then exclude patients < 40 years old *or* >75 years old on test result date

3. Then exclude patients listed as deceased [5] within **60 days after** test result date
4. Then exclude patients with active colon cancer diagnosis [6] within **1 year prior to** test result date
5. Then exclude patients with colectomy [7] **any time prior to and 60 days after** test result date
6. Then exclude patients enrolled in hospice or palliative care [8] within **1 year prior to and 60 days after** test result date
7. Then exclude patients with a diagnosis of pancreatic cancer [9] *or* leukemia (except acute lymphocytic) [10] *or* liver cancer [11] *or* biliary cancer [12] *or* esophageal cancer [13] *or* gastric cancer [14] *or* brain cancer [15] *or* uterine cancer [16] *or* ovarian cancer [17] *or* peritoneal, omental, or mesenteric cancer [18] *or* myeloma [19] *or* lung, bronchus, tracheal, or mesothelial cancer diagnosis [20] within **1 year prior to and 60 days after** test result date
8. Then exclude patients with diagnosis of upper GI bleeding (hematemesis) [21] *or* ulcer of esophagus, stomach or duodenum with bleeding [22] within **6 months prior to** the test result date
9. Then exclude patients with colonoscopy [23] **within 3 years prior to** test result date
10. Then for iron deficiency anemia only, exclude patients with menorrhagia [24] *or* hematuria [25] *or* epistaxis [26] *or* uterine, cervical or vaginal bleeding [27] *or* hemoptysis [28] *or* secondary hemorrhage [29] **within 6 months prior to** test result date
11. Then for iron deficiency anemia only, exclude patients with diagnosis of pregnancy [30] **within 1 year prior to or 60 days after** test result date
12. Then for iron deficiency anemia only, exclude patients with thalassemia [31] **any time prior to or within 60 days after** test result date

Expected Follow-up Criteria

13. Then exclude patients with a completed gastroenterology visit [32] **within 60 days after** test result date
14. Then exclude patients with a colonoscopy [23] performed **within 60 days after** test result date

Footnotes (colorectal)

[1] LOINC: 718-7, 30313-1, 30350-3, 30352-9

[2] LOINC: 30428-7, 787-2

[3] LOINC: 2276-4

[4] LOINC: 50196, 14563, 14564, 14565, 38527, 38526, 57803, 7905, 56490, 56491, 59841, 57804, 2335, 29771, 57804, 59841

[5] Based on status in mortality table

[6] ICD-10 colon: C18.3, C18.4, C18.6, C18.7, C18.0, C18.1, C18.2, C18.5, C18.8, C18.9, C19, C20, C21.1, C21.0, C21.8; ICD-9: 153.xx, 154.0, 154.1, 154.8 (where 'x' is any value between 0 and 9)

[7] ICD-10 colectomy: 0DTE4ZZ, 0DTE0ZZ, 0DTE7ZZ, 0DTE8ZZ; CPT: 44150, 44151, 44155, 44156, 44157, 44158, 44202, 44210, 44211, 44212; ICD-9: 45.81, 45.82, 45.83

[8] ICD-10: Z51.5, or consult code entry for completed hospice/palliative care consult, or consult with primary stop code 351 or 353. ICD-9: V66.7

[9] ICD-10 pancreas: C25.0, C25.1, C25.2, C25.3, C25.4, C25.7, C25.8, C25.9; ICD-9: 157.xx

[10] ICD-10 leukemia: C92.00, C92.4, C92.5, C92.60, C92.01, C92.41, C92.51, C92.02, C92.42, C92.52, C93.00, C93.01, C93.02, C94.00, C94.01, C94.02, C94.20, C94.21, C94.22, C95.00, C95.01, C95.02; ICD-9: 205.0, 206.0, 207.0, 207.2x, 208.0

[11] ICD-10 liver: C22.0, C22.2, C22.3, C22.4, C22.7, C22.8, C22.1, C22.9, C78.7; ICD-9: 155.0, 155.1, 155.2, 197.7

[12] ICD-10 biliary: C23, C24, C24.1, C24.8, C24.9; ICD-9: 156.xx

[13] ICD-10 esophagus: C15.3, C15.4, C15.5, C15.8, C15.9; ICD-9: 150.xx

[14] ICD-10 gastric: C16.0, C16.1, C16.2, C16.3, C16.4, C16.5, C16.6, C16.8, C16.9; ICD-9: 151.xx

[15] ICD-10 brain: C71.0, C71.1, C71.2, C71.3, C71.4, C71.5, C71.6, C71.7, C71.8, C71.9, C79.31, C79.32, C79.49; ICD-9: 191.x, 198.3, 198.4

[16] ICD-10 uterus: C55; ICD-9: 179.xx

[17] ICD-10 ovary: C56.9, C56.1, C56.2; ICD-9: 183.0

[18] ICD-10 peritoneum: C48.1, C45.1, C48.8, C48.2, C78.6; ICD-9: 158.8, 158.9, 197.6

[19] ICD-10 myeloma: C90.00, C90.01, C90.02, D47.Z9; ICD-9: 203.0x, 238.6

[20] ICD-10 lung: C34.0 to C34.3, C34.8, C34.9, C78.00, C78.01, C78.02; ICD-9: 162.0, 162.2x, 162.3x, 162.4x, 162.5x, 162.8x, 162.9x, 163.xx, 197.0, 197.2, 197.3 (where 'x' is any value)

[21] ICD-10 hematemesis: K92.0, K22.11; ICD-9: 578.0

Zimolzak AJ, *et al. BMJ Health Care Inform* 2022; 29:e100565. doi: 10.1136/bmjhci-2022-100565

[22] ICD-10 ulcer: K25.0, K25.1, K25.2, K25.4, K25.6, K26.0, K26.2, K26.4, K26.6, K27.0, K27.2, K27.4, K27.6, K28.0, K28.2, K28.4, K28.6, I85.01, I85.11; ICD-9: 530.21, 531.0x, 531.2x, 531.4x, 531.6x, 532.0x, 532.2x, 532.4x, 532.6x, 533.0x, 533.2x, 533.4x, 533.6x, 534.0x, 534.2x, 534.4x, 534.6x

[23] CPT scope: 44387, 44388, 44389, 44391, 44392, 44394, 45378, 45379, 45380, 45381, 45382, 45383, 45384, 45385, 45386, 45387, 45355, 45391, 45392; ICD-10: 0DJD8ZZ

[24] ICD-10 menorrhagia: N92.0, N92.1, N92.4, N95.0; ICD-9: 626.2, 626.6, 627.0, 627.1

[25] ICD-10 hematuria: R31.9, R31.0, R31.1, R31.2; ICD-9: 599.7x

[26] ICD-10 epistaxis: R04.0; ICD-9: 784.7

[27] ICD-10 uterine: N89.8, N92.5, N93.8; ICD-9: 623.8, 626.8

[28] ICD-10 hemoptysis: R04.2, R04.9, R04.89; ICD-9: 786.3x

[29] ICD-10 secondary: T79.2XXA; ICD-9: 958.2

[30] ICD-10 pregnancy: Z34.00, Z34.80, Z34.90, Z33.1, O09.00, O09.10, O09.291, O09.40, O09.211, O09.30, O09.511, O09.521, O09.611, O09.621, O09.891, O09.892, O09.893, O09.899, O09.90, O09.91, O09.92, O09.93, O00.0, O00.1, O00.2, O00.8, O00.9; ICD-9: 629.81, 631.0, 633.0, 633.01, 633.10, 633.2x, 633.8x, 633.9x, V22.0, V22.1, V22.2, V23.0, V23.1, V23.2, V23.3, V23.41, V23.49, V23.5, V23.7, V23.81, V23.82, V23.83, V23.84, V23.89, V23.9

[31] ICD-10 thalassemia: D56.9, D57.40, D57.419, D56.0, D56.1, D56.2, D56.3, D56.5, D56.8; ICD-9: 282.4x

[32] Based on VA clinic stop code 33, 307, 321, or clinical note title entry for completed GI consult

APPENDIX: Lung Red Flag Criteria

1. Identify all patient records with abnormal chest X-Ray or CT result flagged by radiologist as “suspicious for malignancy” [1]

Clinical Exclusion Criteria

2. Then exclude patients $<$ 18 years old on imaging result date
3. Then exclude patients listed as deceased [2] within **30 days after** imaging result date
4. Then exclude patients with active lung cancer diagnosis [3] within **1 year prior to** imaging result date

5. Then exclude patients with tuberculosis diagnosis [4] within **1 year prior to** and **30 days after** imaging result date
6. Then exclude patients enrolled in hospice or palliative care [5] within **1 year prior to** and **30 days after** imaging result date
7. Then exclude patients with a diagnosis of pancreatic cancer [6] *or* leukemia (except acute lymphocytic) [7] *or* liver cancer [8] *or* biliary cancer [9] *or* esophageal cancer [10] *or* gastric cancer [11] *or* brain cancer [12] *or* uterine cancer [13] *or* ovarian cancer [14] *or* peritoneal, omental, or mesenteric cancer [15] *or* myeloma [16] *or* tracheal cancer diagnosis [17] within **1 year prior to** and **30 days after** imaging result date

Expected Follow-up Criteria

8. Then exclude patients with a repeated chest x-ray or CT [1] **within 30 days after** imaging result date
9. Then exclude patients with a completed PET scan [18] **within 30 days after** imaging result date
10. Then exclude patients with a repeated pulmonary visit [19] **within 30 days after** imaging result date
11. Then exclude patients with a completed thoracic surgery visit [20] **within 30 days after** imaging result date
12. Then exclude patients with a completed multidisciplinary tumor board conference [21] **within 30 days after** imaging result date
13. Then exclude patients with a lung biopsy [22] performed **within 30 days after** imaging result date
14. Then exclude patients with a bronchoscopy [23] performed **within 30 days after** imaging result date
15. Then exclude patients with a lung surgery [24] performed **within 30 days after** imaging result date

Footnotes (lung)

[1] CPT: X-Ray (71010, 71015, 71020, 71021, 71022, 71030, 71035, 71101, 71111); CT (71275, 71250, 71270, 71260)

[2] Based on status in mortality table

[3] ICD-10 lung: C34.00, C34.01, C34.02, C34.10, C34.11, C34.12, C34.2, C34.30, C34.31, C34.32, C34.80, C34.81, C34.82, C34.90, C34.91, C34.92, C78.00, C78.01,

C78.02, C38.4, C45.0, C78.2; ICD-9: 162.2x, 162.3x, 162.4x, 162.5x, 162.8x, 162.9x, 197.0, 163.xx, 197.2 (where ‘x’ is any value)

[4] ICD-10 tuberculosis: A15.0, A15.5, A15.6, A15.7; ICD-9: 010.0x, 010.1x, 010.8x, 010.9x, 011.0x, 011.1x, 011.2x, 011.3x, 011.4x, 011.5x, 011.6x, 011.7x, 011.8x, 011.9x (where ‘x’ is any value between 1 and 6)

[5] ICD-10: Z51.5, or consult code entry for completed hospice/palliative care consult, or consult with primary stop code 351 or 353. ICD-9: V66.7

[6] ICD-10 pancreatic: C25.0, C25.1, C25.2, C25.3, C25.4, C25.7, C25.8, C25.9; ICD-9: 157.xx

[7] ICD-10 leukemia: C92.00, C92.4, C92.5, C92.60, C92.01, C92.41, C92.51, C92.02, C92.42, C92.52, C93.00, C93.01, C93.02, C94.00, C94.01, C94.02, C94.20, C94.21, C94.22, C95.00, C95.01, C95.02; ICD-9: 205.0, 206.0, 207.0, 207.2x, or 208.0

[8] ICD-10 liver: C22.0, C22.2, C22.3, C22.4, C22.7, C22.8, C22.1, C22.9, C78.7; ICD-9: 155.0, 155.1, 155.2, or 197.7

[9] ICD-10 biliary: C23, C24, C24.1, C24.8, C24.9; ICD-9: 156.xx

[10] ICD-10 esophageal: C15.3, C15.4, C15.5, C15.8, C15.9; ICD-9: 150.xx

[11] ICD-10 gastric: C16.0, C16.1, C16.2, C16.3, C16.4, C16.5, C16.6, C16.8, C16.9; ICD-9: 151.xx

[12] ICD-10 brain: C71.0, C71.1, C71.2, C71.3, C71.4, C71.5, C71.6, C71.7, C71.8, C71.9, C79.31, C79.32, C79.49; ICD-9: 191.x, 198.3, or 198.4

[13] ICD-10 uterine: C55; ICD-9: 179.xx

[14] ICD-10 ovarian: C56.9, C56.1, C56.2; ICD-9: 183.0

[15] ICD-10 peritoneal: C48.1, C45.1, C48.8, C48.2, C78.6; ICD-9: 158.8, 158.9, or 197.6

[16] ICD-10 myeloma: C90.00, C90.01, C90.02, D47.Z9; ICD-9: 203.0x, or 238.6

[17] ICD-10 tracheal: C33, C78.39; ICD-9: 162.0, 197.3

[18] CPT: 78811, 78812, 78813, 78814, 78815, 78816, 78810, G0125, G0126, G0210, G0211, G0212, G0213;

[19] Based on VA clinic stop code 312, 104, or clinical note title entry for a completed pulmonary consult

[20] Based on VA clinic stop code 413, 64, or clinical note title entry for a completed thoracic surgery consult

[21] Based on VA clinic stop code 316, or clinical note title entry for a completed tumor board conference consult

[22] **CPT biopsy:** 3162x (where 'x' is any value between 5 and 9), 31633, 31640, 31717, 32400, 32402, 32405, 32098, 32601, 32607, 32608, 32609. **ICD-10 lung biopsy:** 0B9C3ZX, 0B9C4ZX, 0B9C7ZX, 0B9D3ZX, 0B9D4ZX, 0B9D7ZX, 0B9F3ZX, 0B9F4ZX, 0B9F7ZX, 0B9G3ZX, 0B9G4ZX, 0B9G7ZX, 0B9H3ZX, 0B9H4ZX, 0B9H7ZX, 0B9J3ZX, 0B9J4ZX, 0B9J7ZX, 0B9K3ZX, 0B9K4ZX, 0B9K7ZX, 0B9L3ZX, 0B9L4ZX, 0B9L7ZX, 0B9M3ZX, 0B9M4ZX, 0B9M7ZX, 0BBC3ZX, 0BBD3ZX, 0BBF3ZX, 0BBG3ZX, 0BBH3ZX, 0BBJ3ZX, 0BBK3ZX, 0BBL3ZX, 0BBM3ZX, 0B9K8ZX, 0B9L8ZX, 0B9M8ZX, 0BBK7ZX, 0BBK8ZX, 0BBL7ZX, 0BBL8ZX, 0BBM4ZX, 0BBM7ZX, 0BBM8ZX, 0B9K0ZX, 0B9L0ZX, 0B9M0ZX, 0BBK0ZX, 0BBL0ZX, 0BBM0ZX. **ICD-10 bronchus biopsy:** 0B933ZX, 0B934ZX, 0B937ZX, 0B938ZX, 0B943ZX, 0B944ZX, 0B947ZX, 0B948ZX, 0B953ZX, 0B954ZX, 0B957ZX, 0B958ZX, 0B963ZX, 0B964ZX, 0B967ZX, 0B968ZX, 0B973ZX, 0B974ZX, 0B977ZX, 0B978ZX, 0B983ZX, 0B984ZX, 0B987ZX, 0B988ZX, 0B993ZX, 0B994ZX, 0B997ZX, 0B998ZX, 0B9B3ZX, 0B9B4ZX, 0B9B7ZX, 0B9B8ZX, 0BB33ZX, 0BB34ZX, 0BB37ZX, 0BB38ZX, 0BB43ZX, 0BB44ZX, 0BB47ZX, 0BB48ZX, 0BB53ZX, 0BB54ZX, 0BB57ZX, 0BB58ZX, 0BB63ZX, 0BB64ZX, 0BB67ZX, 0BB68ZX, 0BB73ZX, 0BB74ZX, 0BB77ZX, 0BB78ZX, 0BB83ZX, 0BB84ZX, 0BB87ZX, 0BB88ZX, 0BB93ZX, 0BB94ZX, 0BB97ZX, 0BB98ZX, 0BBB3ZX, 0BBB4ZX, 0BBB7ZX, 0BBB8ZX, 0B930ZX, 0B940ZX, 0B950ZX, 0B960ZX, 0B970ZX, 0B980ZX, 0B990ZX, 0B9B0ZX, 0BB30ZX, 0BB40ZX, 0BB50ZX, 0BB60ZX, 0BB70ZX, 0BB80ZX, 0BB90ZX, 0BBB0ZX. **ICD-10 pleural biopsy:** 0BBC4ZX, 0BBD4ZX, 0BBF4ZX, 0BBG4ZX, 0BBH4ZX, 0BBJ4ZX, 0BBK4ZX, 0BBL4ZX, 0B9N0ZX, 0B9N3ZX, 0B9N4ZX, 0B9P0ZX, 0B9P3ZX, 0B9P4ZX, 0BBN0ZX, 0BBN3ZX, 0BBP0ZX, 0BBP3ZX, 0W990ZX, 0W993ZX, 0W994ZX, 0W9B0ZX, 0W9B3ZX, 0W9B4ZX. **ICD-10 chest biopsy:** 0W980ZX, 0W983ZX, 0W984ZX, 0WB80ZX, 0WB83ZX, 0WB84ZX, 0WB8XZX. **ICD-10 mediastinum biopsy:** 0W9C3ZX, 0W9C4ZX, 0WBC3ZX, 0WBC4ZX. **ICD-9:** 33.24, 33.25, 33.26, 33.27, 33.28 (where 'x' is any value between 4 and 8), 34.20, 34.23, 34.24, 34.25.

[23] **CPT bronchoscopy:** 3162x (where 'x' is any value between 1 and 4), 3163x (where 'x' is any value between 0 and 8), 31641, 31643, 31645, 31646, 31647, 31648, 31649, 31650, 31651, 31656, 31659, 31660, 31661, 31725, 32035. **ICD-10:** 0BBN4ZX, 0BBP4ZX, 0BJ08ZZ, 0WJQ4ZZ, 0WJC4ZZ, 0BJ08ZZ, 0BJK8ZZ, 0BJL8ZZ. **ICD-9:** 33.20, 33.21, 33.22, 33.23.

[24] **CPT surgery:** 32036, 32095, 32096, 32097, 32100, 32120, 32140, 32141, 32150, 32200, 32201, 32310, 32315, 32320, 32440, 32442, 32445, 32450, 32480, 32482, 32484, 32485, 32486, 32488, 32490, 32491, 32500, 32503, 32504, 32505, 32520, 32522, 32525, 32540, 32545, 32656, 32657, 32663, 32666, 32667, 32668, 32669, 32670, 32671, 32672, 32700, 32705. **ICD-10 ablation:** 0B5K0ZZ, 0B5L0ZZ, 0B5M0ZZ, 0B5K3ZZ, 0B5L3ZZ, 0B5M3ZZ, 0B5K4ZZ, 0B5L4ZZ, 0B5M4ZZ, 0B5K7ZZ, 0B5K8ZZ, 0B5L7ZZ, 0B5L8ZZ, 0B5M7ZZ, 0B5M8ZZ. **ICD-10 resection:** 0B5K8ZZ, 0B5L8ZZ, 0B5M8ZZ, 0BBK8ZZ, 0BBL8ZZ, 0BBM4ZZ, 0BBM8ZZ. **ICD-10 lobectomy:** 0BTC4ZZ, 0BTD4ZZ, 0BTF4ZZ, 0BTG4ZZ, 0BTJ4ZZ, 0BTC0ZZ, 0BTD0ZZ, 0BTF0ZZ, 0BTG0ZZ, 0BTJ0ZZ.

ICD-10 thoracotomy: 02JA0ZZ, 0WJC0ZZ. **ICD-10 thoracoscopy:** 0BJ04ZZ, 0WJQ4ZZ. **ICD-10 excision:** 0BBK4ZZ, 0BBL4ZZ, 0B5K0ZZ, 0B5K3ZZ, 0B5K7ZZ, 0B5L0ZZ, 0B5L3ZZ, 0B5L7ZZ, 0B5M0ZZ, 0B5M3ZZ, 0B5M7ZZ, 0BBK0ZZ, 0BBK3ZZ, 0BBK7ZZ, 0BBL0ZZ, 0BBL3ZZ, 0BBL7ZZ, 0BBM0ZZ, 0BBM3ZZ, 0BBM7ZZ, 0BBC4ZZ, 0BBD4ZZ, 0BBF4ZZ, 0BBG4ZZ, 0BBH4ZZ, 0BBJ4ZZ, 0BBK4ZZ, 0BBL4ZZ, 0BTH4ZZ, 0BBK0ZZ, 0BBK3ZZ, 0BBK7ZZ, 0BBL0ZZ, 0BBL3ZZ, 0BBL7ZZ. **ICD-10 pneumonectomy:** 0BTK4ZZ, 0BTL4ZZ, 0BTM4ZZ. 0BTK0ZZ, 0BTL0ZZ, 0BTM0ZZ. **ICD-10 bronchus excision:** 0B534ZZ, 0B538ZZ, 0B544ZZ, 0B548ZZ, 0B554ZZ, 0B558ZZ, 0B564ZZ, 0B568ZZ, 0B574ZZ, 0B578ZZ, 0B584ZZ, 0B588ZZ, 0B594ZZ, 0B598ZZ, 0B5B4ZZ, 0B5B8ZZ, 0BB34ZZ, 0BB38ZZ, 0BB44ZZ, 0BB48ZZ, 0BB54ZZ, 0BB58ZZ, 0BB64ZZ, 0BB68ZZ, 0BB74ZZ, 0BB78ZZ, 0BB84ZZ, 0BB88ZZ, 0BB94ZZ, 0BB98ZZ, 0BBB4ZZ, 0BBB8ZZ, 0B530ZZ, 0B533ZZ, 0B537ZZ, 0B540ZZ, 0B543ZZ, 0B547ZZ, 0B550ZZ, 0B553ZZ, 0B557ZZ, 0B560ZZ, 0B563ZZ, 0B567ZZ, 0B570ZZ, 0B573ZZ, 0B577ZZ, 0B580ZZ, 0B583ZZ, 0B587ZZ, 0B590ZZ, 0B593ZZ, 0B597ZZ, 0B5B0ZZ, 0B5B3ZZ, 0B5B7ZZ, 0BB30ZZ, 0BB33ZZ, 0BB37ZZ, 0BB40ZZ, 0BB43ZZ, 0BB47ZZ, 0BB50ZZ, 0BB53ZZ, 0BB57ZZ, 0BB60ZZ, 0BB63ZZ, 0BB67ZZ, 0BB70ZZ, 0BB73ZZ, 0BB77ZZ, 0BB80ZZ, 0BB83ZZ, 0BB87ZZ, 0BB90ZZ, 0BB93ZZ, 0BB97ZZ, 0BBB0ZZ, 0BBB3ZZ, 0BBB7ZZ, 0BT30ZZ, 0BT34ZZ, 0BT40ZZ, 0BT44ZZ, 0BT50ZZ, 0BT54ZZ, 0BT60ZZ, 0BT64ZZ, 0BT70ZZ, 0BT74ZZ, 0BT80ZZ, 0BT84ZZ, 0BT90ZZ, 0BT94ZZ, 0BTB0ZZ. **ICD-9:** 32.0, 32.01, 32.09, 32.1, 32.20, 32.23, 32.34, 32.25, 32.26, 32.28, 32.29, 32.3, 32.39, 32.4, 32.41, 32.49, 34.02, 34.21, 32.5, 32.59.
